# Reconstruction of the Adult Hemifacial Microsomia Patient with Temporomandibular Joint Total Joint Prosthesis and Orthognathic Surgery

**DOI:** 10.1155/2018/2968983

**Published:** 2018-03-15

**Authors:** Piero Cascone, Valentino Vellone, Valerio Ramieri, Emanuela Basile, Achille Tarsitano, Claudio Marchetti

**Affiliations:** ^1^Sapienza Università di Roma, Via del Policlinico, Roma, Italy; ^2^Department of Biomedical and Neuromotor Sciences, Maxillofacial Surgery Unit, S. Orsola-Malpighi Hospital, Alma Mater Studiorum University of Bologna, Bologna, Italy

## Abstract

**Background:**

HFM patients' reconstruction has always been a challenge for maxillofacial surgeons, and numerous reconstructive techniques have been described. Surgical treatment depends on the patient's age and contemplates Temporomandibular Joint (TMJ) reconstruction in conjunction with orthognathic surgery, usually necessary following completion of growth to maximize the functional and esthetic results. Distraction osteogenesis had gained popularity as valid alternative in growing patients, but the two primary methods to reconstruct the TMJs involve the use of autogenous, using free or microvascular bone grafts, or alloplastic graft, but there is no widely accepted method.

**Methods:**

The increasing use of temporomandibular prosthesis for temporomandibular problems has led us to use them even in HFM. A case of female nongrowing patients with HFM type IIb treated with temporomandibular prosthesis in an all-in-one protocol is presented.

**Results:**

Incisal opening, measured with BioPAK system (Bioresearch Inc., Milwaukee, USA), was 21.4 mm in the presurgical period and 32.2 mm after all-in-one procedure, for an increase of 50.5%. Excursive movement to the right side was 2.2 mm in the presurgical period and was 1.5 mm after surgery, for a decrease of 31.8%. Left excursion movement changed from 5 mm to 6.1 mm, for an increase of 22.0%.

**Conclusions:**

The TMJ Concepts patient-fitted TJP in conjunction with orthognathic surgery for TMJ and jaw reconstruction is a valid option for patients with HFM.

## 1. Introduction

Hemifacial microsomia (HFM) is the second most common facial birth defect after cleft lip and palate. The estimated incidence is of 1 of every 4000 to 5600 live births.

The aetiology and pathogenesis of hemifacial microsomia are still unknown.

The two proposed etiopathogenic theories include hematoma of the stapedial artery during fetal development and abnormal neural crest migration. Both models explain the variable and asymmetric nature of this condition.

The clinical presentation is variable, and many classification systems have been developed.

Figueroa and Pruzansky [1], subsequently modified by Vento et al. [2], described three mandibular types. In type I, the temporomandibular joint and ramus are well formed but smaller than normal. In type II, the temporomandibular joint, ramus, and glenoid fossa are hypoplastic and malformed. In type III, the entire ramus is missing.

Kaban et al. [3] added a subdivision of type II, identifying type IIa as a ramus with abnormal size and shape and type IIb as a ramus and TMJ with abnormal size, shape, and function. This classification system may be the most useful to the surgeon in the preoperative evaluation because of its simplicity and inclusion of the TMJ anatomy and function.

HFM patients' reconstruction has always been a challenge for maxillofacial surgeons, and numerous reconstructive techniques have been described.

Surgical treatment depends on the patient's age and contemplates TMJ reconstruction in conjunction with orthognathic surgery, usually necessary following completion of growth to maximize the functional and esthetic results [4].

TMJ and jaw reconstruction in patients with HFM has been described using autogenous tissue such as rib graft or sternoclavicular graft alone or in conjunction with orthognathic surgery in patients with various degrees of deformity, but without high-quality outcomes documentation.

It must be remembered that most of the patients reconstructed with an autogenous graft require at least one additional maxillomandibular surgical procedure due to the high percentage of ankylosis, fracture, variable growth behavior, resorption, infection, and donor side morbity [5].

Another option for early intervention is the use of distraction osteogenesis but requires multiple operations to place and remove the distractors, and this can lead to difficulty in controlling the vector of growth.

Improving the predictability of results and limiting correction of the jaw and TMJ deformities to 1, major operation can best be achieved by waiting until growth is complete, provided that the patient is functionally and psychologically stable [4].

The development of custom-fitted total joint prosthesis (TJP) provides an additional option to aid in reconstruction of the HFM patient in conjunction with orthognathic surgical procedures [6].

A case of female nongrowing patient with HFM type IIb treated with temporomandibular prosthesis in an all-in-one protocol is presented.

## 2. Case Description

A 22-year-old female with left side hemifacial microsomia (type IIb) was referred to the Maxillo-Facial Surgery Department at the Sapienza Università di Roma for evaluation and treatment.

Clinical examination and 3-dimensional CT scans showed unilateral hypoplasia of the mandibular condyle, ramus, and body; retrognathic mandible deviated toward the ipsilateral side; large occlusal plane angle with a hyperdivergent facial morphology; hypoplasia of the ipsilateral maxilla and temporal bone; Class II skeletal and Class II occlusal relation; canting of the occlusal plane; ear anomalies and ipsilateral soft tissue deficiency affecting skin, subcutaneous, muscular, and glandular tissues; decreased ipsilateral facial height.

Following completion of clinical, cephalometric, dental model, and radiographic imaging analyses, the treatment plan included the following:

1. Presurgical orthodontics to align and level the maxillary and mandibular arches.
2. Surgery
  - Right mandibular ramus sagittal split osteotomy to advance the mandible in a counterclockwise rotation.
  - Left TMJ reconstruction to advancement the mandible in a counterclockwise direction with custom-fitted total joint prostheses (TMJ Concepts, Ventura, California, USA).
  - Maxillary osteotomies to advance the maxilla in a counterclockwise direction and level the occlusal plane transversely with rigid fixation and bone grafting.
3. Postsurgical orthodontics to refine and retain the occlusion.

A CT scan was acquired, and a 3-D stereolithic model was produced for a better evaluation and to simulate subsequent mandibular surgery.

**Figure 1:**
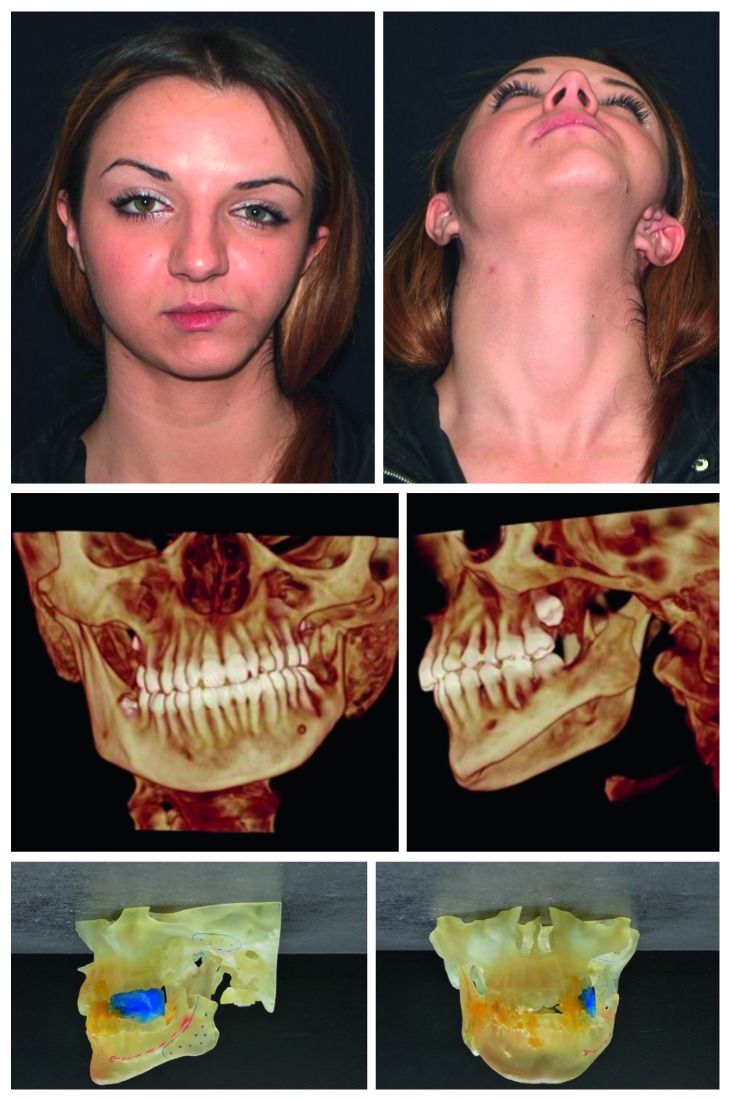
Patient presurgery in frontal view and axial view. 3D CT scan pre surgery in frontal and left lateral views. 3-dimensional plastic model of the patient's jaws, TMJs, and cranial base structures repositioned and fixed with quick-cure acrylic.

The bone thickness, confirmed by CT scan (Figure 1), in temporal root of the zygomatic arch and glenoid fossa were adequate for the fixation of the fossa component of the prosthesis.

The custom-made prosthesis was developed basing on 3-dimensional virtual program (Dolphin 11.5, Dolphin Imaging and Management Solutions, Chatsworth, CA, USA) and stereolithographic model.

The sagittal split osteotomy was performed to reposition the mandible in the predetermined new position based on the clinical evaluation, dental model analysis, and the prediction tracing.

The mandible was moved downward in the back, advanced in a counterclockwise direction, transversely levelled and fixed with quick-cure acrylic (Figure 1).

A LeFort I osteotomy was performed to correct the canting.

A wax disc was recreated to reconstruct as much as possible the vertical dimension after the sagittal split osteotomy.

Before surgery, dental models mounted on an anatomic articulator were used to replicate the mandibular repositioning performed on the 3-dimensional plastic model for the construction of the intermediate surgical occlusal splint necessary for precise positioning of the mandible at surgery.

Intermediate splint construction was required to improve the accuracy of a patient's surgery and outcome according to “mandible first” surgical sequence.

### 2.1. Surgical Technique

In the operating room, the same time of model surgery was followed.

Sagittal split osteotomy on the right side was performed.

The left side was approached through an external preauricular and submandibular incision with reflection of the musculature, and mobilization of the mandible.

The dissection was carried down to the temporal bone anterior to the internal meatus and extended medially. A subperiosteal tunnel was created over the hypoplastic ramus to connect to the base of the temporal bone.

The TMJ fossa component of the TJP was placed and secured to the temporal bone with screws.

The mandibular component was inserted through the submandibular incision, and the condyle was seated into the fossa component and secured to the ramus with bicortical screws. The incisions were closed.

A separate set of instruments was used to maintain sterility of the TMJ area.

Moreover, surgical gloves were changed every time that surgeons passed from mouth to TMJ area.

A prefabricated intermediate occlusal splint was placed in order to position the mandible into its final predetermined position.

Intermaxillary fixation was performed, and mandibular sagittal split osteotomy stabilized with bicortical screws, and the oral incision closed.

The IMF and occlusal splint were removed. Through a maxillary vestibular incision, the maxillary osteotomies were completed. The maxilla was downfractured, mobilized 6 millimetres down in the left side and fixed with four bone plates, with 2 screws above and 2 screws below the osteotomy site for each plate. A laminar, cortical, heterologous bone graft (Tecnoss Medical Devices, Giaveno, Italy) was used to fill the osteotomy's gap.

The final occlusion was established and secured with IMF.

As refinements, genioplasty and lipofilling procedure was performed.

**Figure 2:**
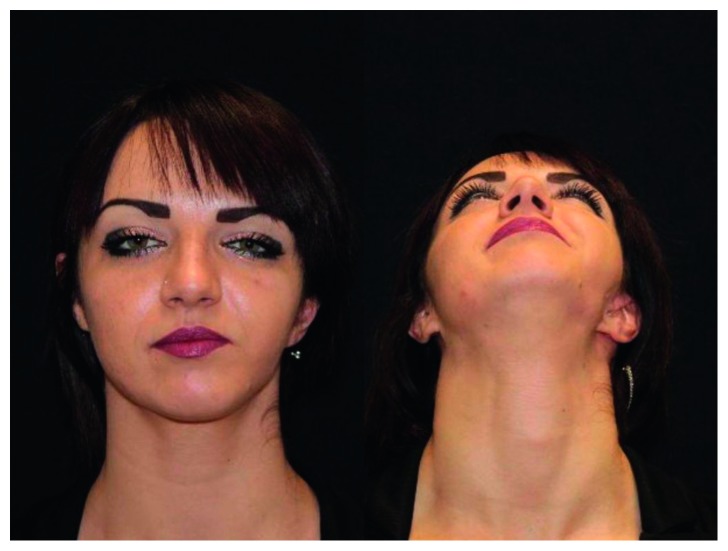
Patient 1 year after surgery in frontal and axial views.

**Figure 3:**
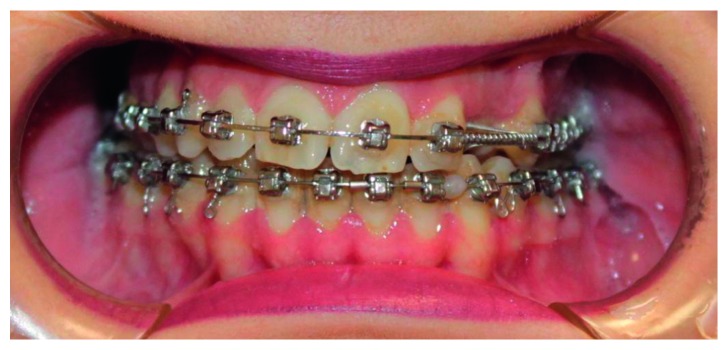
Patient occlusion 1 year after surgery.

**Figure 4:**
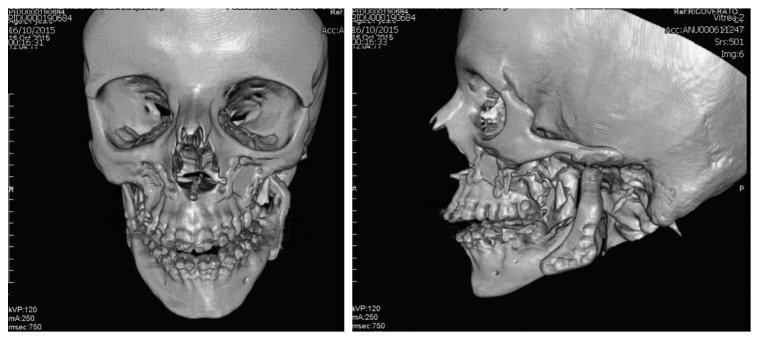
3D CT scan after surgery in frontal and lateral views.

The IMF was removed, and light elastics were applied to guide the occlusion and decrease the stress on the muscles of mastication (Figures 2–4).

## 3. Results

Incisal opening, measured with BioPAK system (Bioresearch Inc., Milwaukee, USA), was 21.4 mm in the presurgical period and 32.2 mm after 1-year follow-up, for an increase of 50.5%. Excursive movement to the right side was 2.2 mm in the presurgical period and was 1.5 mm after surgery, for a decrease of 31.8%. Left excursion movement changed from 5 mm to 6.1 mm, for an increase of 22.0%.

## 4. Discussion

Management of hemifacial microsomia is controversial. Numerous reconstructive techniques have been described.

Early surgical intervention to lengthen the ramus on the affected side has often been advocated as a method to prevent maxillary compensatory deformity.

Unfortunately, mandibular distraction osteogenesis requires multiple operations to place and remove the distractors, and this can lead to difficulty in controlling the vector of growth; furthermore, very few long-term reports are available.

Anyway, the two primary methods to reconstruct the TMJs involve the use of autogenous tissues (i.e., rib or sternoclavicular grafts (SCGs)) versus alloplastic total joint prosthetic devices.

Autologous bone grafting techniques are well established and accepted. A costochondral bone graft is the most commonly used autogenous replacement for mandibular ramus/condyle defects, especially in growing children.

Theoretically, the costochondral grafts keep a “growth potential” but has been shown to be unpredictable or resulting in ankyloses, due to allograft and/or fixation failure or for an uncooperative patient nature with postreconstruction physical therapy.

Recent studies have even questioned the necessity for using a cartilaginous graft to restore and maintain mandibular growth.

Peltomäki et al. [7] in investigations of mandibular growth after costochondral grafting supported previous experiments with regard to the inability of the graft to adapt to the growth velocity of the new environment. If autogenous grafts fail to incorporate into the host bone, fail to grow, grow horizontally rather than vertically, or more commonly become ankylosed; under these circumstances, the patient basically becomes disabled—unable to eat foods because they cannot open their mouths wide enough. These and other issues can lead to social ostracization, depression, and very poor life quality.

Improving the predictability of results and limiting correction of the jaw and TMJ deformities to 1, major operation can best be achieved by waiting until growth is complete, provided that the patient is functionally and psychologically stable [4].

The introduction of alloplastic TJR has improved the quality of life for many adult orthopedic and TMJ patients with unsalvageable functional and anatomical joint pathology [8, 9]. However, these devices, because they are a biomechanical rather than biological nature, have a finite life-span making them an unattractive alternative in the growing patient.

Taking into consideration the information available for autogenous tissue grafting, the use of alloplastic TMJ TJR may be an option in type II HFM onwards [10].

A further advantage for TJR is the possibility, when zygomatic arch and glenoid fossa are absent, to plan the fossa component custom-fitted to the base of the skull and lateral temporal bone morphology.

Correction of the transversal anomaly of HFM has always been difficult because of deficiencies of facial skeleton and the overlying soft tissue.

The concept and technique of using TMJ TJP for TMJ reconstruction and simultaneous mandibular advancement with counterclockwise rotation of the maxillomandibular complex was developed by Wolford et al. in 1990 and was first used for HFM in 1997 [6].

This operation permits to solve many HFM-related problems at one time while multiple TMJ operations create scar tissue and interrupt normal blood flow and normal physiologic nutritional distribution to the anatomic structures.

Concern has been raised about the longevity of TMJ TJR devices.

The longevity of prosthesis for any joint is dependent on materials, design, stability, and functional loading.

It is difficult to determine the functional load for the TMJ prosthesis. For the average adult, the biting forces generated at the molars is approximately 60 pounds (27 kg) and that for the incisors is 35 pounds (15 kg) [11].

Although the life expectancy of TMJ Concepts TMJ TJR devices is still unknown [12], it should be longer than their orthopedic joint counterparts.

Potential risks and concerns using TJPs include (1) an unknown functional service life of the TMJ Concepts TJP; (2) surgical risks associated with TMJ reconstruction; (3) infection; and (4) development of hypersensitivity to the materials in the prostheses.

## 5. Conclusions

The TMJ Concepts patient-fitted TJP in conjunction with orthognathic surgery for TMJ and jaw reconstruction is a valid option for patients with HFM because (1) no bone graft donor site is required; (2) it does not require reconstruction of the glenoid fossa; (3) it is a patient-fitted device to meet a patient's specific anatomic requirements for mandibular advancement, vertical lengthening, and TMJ reconstruction; and (4) treatment results are highly predictable and stable in relation to skeletal and occlusal stability, TMJ function, improved facial balance, and comfort.
